# Longitudinal Swallowing and Salivary Changes With CLR 131 and Re‐Irradiation in Recurrent Head and Neck Cancer

**DOI:** 10.1002/hed.70309

**Published:** 2026-05-15

**Authors:** Jenni Wu, Sara Gustafson, Meredith Hyun, Roxana Alexandridis, Mark Nicosia, Timothy McCulloch, Justine Bruce, Randall Kimple, Nicole Rogus‐Pulia

**Affiliations:** ^1^ Department of Communication Sciences and Disorders University of Wisconsin‐Madison Madison Wisconsin USA; ^2^ Department of Medicine School of Medicine and Public Health, University of Wisconsin‐Madison Madison Wisconsin USA; ^3^ Geriatric Research Education and Clinical Center, William S. Middleton Memorial Veterans Hospital Madison Wisconsin USA; ^4^ Center for Health Disparities Research, University of Wisconsin‐Madison Madison Wisconsin USA; ^5^ Department of Biostatistics and Medical Informatics School of Medicine and Public Health Madison Wisconsin USA; ^6^ Widener University Chester Pennsylvania USA; ^7^ Department of Surgery School of Medicine and Public Health, University of Wisconsin‐Madison Madison Wisconsin USA; ^8^ Department of Hematology, Medical Oncology, and Palliative Care University of Wisconsin‐Madison Madison Wisconsin USA; ^9^ Department of Human Oncology University of Wisconsin‐Madison Madison Wisconsin USA

## Abstract

**Background:**

Patients with recurrent head and neck cancer (HNC) often present with severe, persistent dysphagia and xerostomia following prior chemoradiation. Although swallowing impairments and salivary dysfunction have been reported in this population, prior longitudinal studies have not examined changes in salivary composition or how these changes relate to swallowing physiology.

**Purpose:**

This study characterized longitudinal changes in swallowing and salivary function—including composition—following treatment with CLR 131, a novel tumor‐selective radiotherapeutic, combined with external beam radiation therapy (EBRT).

**Methods:**

Twelve patients with locoregionally recurrent HNC demonstrating CLR 131 uptake on SPECT/CT imaging were enrolled. Videofluoroscopic swallowing (VFS) studies and stimulated saliva collections were performed at baseline, 3, 6, and 12 months posttreatment. Outcomes included Dynamic Imaging Grade of Swallowing Toxicity (DIGEST) scores, swallowing temporal measures, patient‐reported dysphagia using Eating Assessment Tool (EAT‐10), and salivary flow rate, pH, and extensional viscosity.

**Results:**

At baseline, participants demonstrated significantly worse swallowing and salivary function than published normative data (*p* < 0.001). Most swallowing measures remained stable following treatment; however, time to laryngeal vestibule closure with 5 mL pudding was prolonged at 3 months (*p* < 0.02). Stimulated salivary flow rate declined significantly at 12 months (*p* < 0.001), while pH decreased at 3 months (*p* < 0.05) and extensional viscosity increased at 6 months (*p* < 0.02). From baseline to 3 months, EAT‐10 scores were strongly associated with salivary pH and viscosity changes (*r* = 0.84 and −0.99, respectively). No swallowing timing measures predicted DIGEST safety or efficiency grades.

**Conclusions:**

These findings suggest that CLR 131 + EBRT did not exacerbate objective swallowing impairment in the early post‐treatment period, but patient‐reported dysphagia was sensitive to post‐treatment salivary alterations. Integrating salivary assessments with instrumental and patient‐reported outcomes may enhance detection of functional toxicity and guide targeted dysphagia management in recurrent HNC.

## 1 Background

Head and neck cancer (HNC) is the seventh most common cancer worldwide, accounting for more than 660,000 new cases and 325 000 deaths annually [1]. HNC encompasses malignancies of the oropharynx, nasopharynx, larynx, oral cavity, and salivary glands. The disease significantly impacts quality of life, as patients often experience persistent and severe physical, functional, and psychosocial challenges [2]. One of the most common and debilitating morbidities associated with HNC is dysphagia, or difficulty swallowing, which impairs nutritional intake, social engagement, and increases risk of aspiration‐related complications [2], including pneumonia. Dysphagia is prevalent in 71.3% of patients undergoing chemoradiation and in 40.3% of those treated with radiation therapy (RT) alone [3].

RT remains a cornerstone of HNC management and is often combined with other forms of treatment, including chemotherapy, surgery, and/or immunotherapy. While modern conformal techniques have improved tumor control and reduced certain toxicities [4, 5, 6, 7], late radiation‐associated dysphagia (late‐RAD) and other adverse effects, including mucositis, xerostomia, fibrosis, and trismus remain prevalent [8, 9, 10]. Xerostomia (dry mouth) and hyposalivation (reduction in saliva production) are frequent and persistent side effects of RT that can negatively impact swallowing as saliva plays a critical role in facilitating bolus formation, cohesion and breakdown [9, 10, 11, 12]. Prior work across multiple patient populations (HNC, Sjogren's syndrome, acute stroke, Parkinson's disease) has demonstrated that various salivary changes (e.g., reduced salivary flow rate, decreased salivary neuropeptide; substance P levels [13, 14, 15]) are associated with swallowing impairments. Individuals with reduced salivary flow rate, including those with HNC [16] and Sjogren's syndrome [17], have been shown to demonstrate changes in swallowing biomechanics, such as longer oral transit and increased pharyngeal residue.

Despite advances in technology for RT delivery, about half of individuals with advanced HNC will experience locoregional recurrence, which is typically managed with salvage surgery and/or re‐irradiation (re‐RT) [18], with limited success. Retreatment is necessary for some patients but often carries a substantial risk of lasting damage to surrounding tissues, including complications such as cognitive impairment, hypothyroidism, and osteoradionecrosis [6, 7, 19, 20, 21]. Moreover, alternative treatment options are frequently limited—surgery may be hindered by tumor involvement of critical structures, and re‐RT by the tolerance of normal tissue. A new CLR 131 [18‐(p‐[131I]‐iodophenyl) octadecyl phosphocholine] is an investigational radio‐iodinated cancer therapeutic that exploits the affinity of malignant cells for phospholipid ethers, enabling selective delivery of radiation to tumor cells. When paired with external‐beam radiation therapy (EBRT), CLR 131 may allow lower external radiation doses, thereby sparing healthy structures and reducing toxicity. While CLR 131 has demonstrated antitumor activity in glioma, multiple myeloma, and other various tumor models (e.g., breast, prostate, colorectal, ovarian, renal, pancreatic, and lung) [22], its effects on swallowing and salivary function in recurrent HNC have not yet been studied. Understanding the changes in swallowing and salivary function in recurrent HNC after CLR 131 and EBRT will help develop less toxic re‐RT protocols and guide treatment to preserve functional outcomes.

Therefore, this study aimed to (1) characterize swallowing and salivary function in a cohort of patients undergoing oncologic treatment for recurrent HNC, relative to normative data; (2) evaluate longitudinal changes in swallowing and salivary outcomes following CLR 131 and EBRT; (3) determine the impact of swallowing function and salivary characteristics on patient‐reported dysphagia burden; and (4) identify physiological predictors of swallowing safety and efficiency using a validated outcome measurement, Dynamic Imaging Grade of Swallowing Toxicity (DIGEST), following treatment. Together, these aims were designed to provide a comprehensive assessment of the effects of CLR 131 with EBRT on swallowing and salivary outcomes in patients with recurrent HNC. This longitudinal study is one of the first to examine relationships among salivary composition and swallowing dynamics in this population.

## 2 Methods

### 2.1 Study Sample and Setting

The present study was a secondary analysis of data generated through a prospective, single center, open‐label clinical trial that evaluated the feasibility and tolerability of CLR 131 in combination with EBRT in patients with locoregionally recurrent HNC [23]. Swallowing and salivary function were assessed as part of this prospective study. Participants were enrolled through the University of Wisconsin Carbone Cancer Center.

Inclusion criteria for enrollment in the clinical trial were histologically confirmed recurrent or metastatic HNC, locoregional recurrence deemed suitable for EBRT, and CLR 131 uptake at the recurrence site via single‐photon emission computed tomography with computed tomography (SPECT/CT) imaging. Exclusion criteria were total laryngectomy, extradural disease in contact with the spinal cord, where radiation‐induced swelling could risk spinal cord compression, or chronic immunosuppression equivalent to > 10 mg prednisone daily.

The study protocol was approved by the University of Wisconsin‐Madison's Institutional Review Board (2019‐0681). All participants provided written consent.

### 2.2 Treatment Protocol

All participants underwent screening and procedures for CLR 131 and EBRT. Dosing was determined by multidisciplinary tumor board consensus. A dosimetry test dose of CLR 131 (10–15 mCi) was administered to confirm tumor uptake. Upon confirmation, participants received two intravenous doses of CLR 131 (Day 1 and Day 8). SPECT/CT was performed on Days 2–8 to assess absorbed dose distribution. EBRT was administered unless personalized dose calculations indicated that two CLR 131 doses exceeded 60 Gy.

### 2.3 Assessments

Swallowing and saliva assessments were administered at baseline as well as 3, 6, and 12 months posttreatment. None of the participants received swallowing or salivary treatment. Table 1 lists all measures and definitions.

**TABLE 1:** List of all measures and their definitions.

Category	Measure	Definition	Baseline	3 months	6 months	12 months
Swallowing measures	DIGEST safety	Severity of airway invasion	*N* = 11	*N* = 7	*N* = 6	*N* = 0
	DIGEST efficiency	Severity of pharyngeal residue	*N* = 11	*N* = 7	*N* = 6	*N* = 0
	DIGEST overall	Severity of combined airway invasion and pharyngeal residue	*N* = 11	*N* = 7	*N* = 6	*N* = 0
	Swallow reaction time	Interval between bolus passing mandible and hyoid burst (ms)	*N* = 8 (thin) *N* = 10 (pudding)	*N* = 7 (thin) *N* = 7 (pudding)	*N* = 3 (thin) *N* = 5 (pudding)	*N* = 0 (thin) *N* = 0 (pudding)
	Hyoid burst to UES opening	Interval between hyoid burst and UES opening (ms)	*N* = 10 (thin) *N* = 10 (pudding)	*N* = 8 (thin) *N* = 7 (pudding)	*N* = 5 (thin) *N* = 5 (pudding)	*N* = 0 (thin) *N* = 0 (pudding)
	UES opening duration	Interval between UES opening and UES closure (ms)	*N* = 10 (thin) *N* = 10 (pudding)	*N* = 8 (thin) *N* = 7 (pudding)	*N* = 5 (thin) *N* = 5 (pudding)	*N* = 0 (thin) *N* = 0 (pudding)
	Time to LVC	Interval between hyoid burst and laryngeal vestibule closure (ms)	*N* = 10 (thin) *N* = 10 (pudding)	*N* = 8 (thin) *N* = 7 (pudding)	*N* = 5 (thin) *N* = 5 (pudding)	*N* = 0 (thin) *N* = 0 (pudding)
	LVC duration	Interval between laryngeal vestibule closure and laryngeal vestibule closure off (ms)	*N* = 10 (thin) *N* = 10 (pudding)	*N* = 8 (thin) *N* = 7 (pudding)	*N* = 5 (thin) *N* = 5 (pudding)	*N* = 0 (thin) *N* = 0 (pudding)
	Vallecular residue	Amount of residue in valleculae normalized to C2–C4 area (%)	*N* = 10 (thin) *N* = 10 (pudding)	*N* = 8 (thin) *N* = 7 (pudding)	*N* = 5 (thin) *N* = 5 (pudding)	*N* = 0 (thin) *N* = 0 (pudding)
	Pyriform sinus residue	Amount of residue in pyriform sinus normalized to C2–C4 area (%)	*N* = 10 (thin) *N* = 10 (pudding)	*N* = 8 (thin) *N* = 7 (pudding)	*N* = 5 (thin) *N* = 5 (pudding)	*N* = 0 (thin) *N* = 0 (pudding)
	EAT‐10 score	Total score from EAT‐10	*N* = 12	*N* = 10	*N* = 5	*N* = 2
Salivary measures	Salivary flow rate	Volume of saliva collected over 5 min during mastication at 70 bpm (mL/min)	*N* = 11	*N* = 8	*N* = 5	*N* = 2
	Salivary pH	Acidity or alkalinity of saliva on logarithmic scale	*N* = 11	*N* = 8	*N* = 5	*N* = 2
	Salivary extensional viscosity	Extensional viscosity measured using CaBER (Pa·s)	*N* = 6 (9.0–9.5 *ε*) *N* = 5 (9.5–10.0 *ε*) *N* = 6 (9.0–10.0 *ε*)	*N* = 4 (9.0–9.5 *ε*) *N* = 2 (9.5–10.0 *ε*) *N* = 4 (9.0–10.0 *ε*)	*N* = 2 (9.0–9.5 *ε*) *N* = 2 (9.5–10.0 *ε*) *N* = 2 (9.0–10.0 *ε*)	*N* = 1 (9.0–9.5 *ε*) *N* = 1 (9.5–10.0 *ε*) *N* = 1 (9.0–10.0 *ε*)
	Salivary break up time	Time it takes for sample to break (s)	*N* = 10	*N* = 8	*N* = 4	*N* = 1

Abbreviations: CaBER, capillary breakup extensional rheometer; DIGEST, Dynamic Imaging Grade of Swallowing Toxicity; EAT‐10, Eating Assessment Tool; LVC, laryngeal vestibule closure; UES, upper esophageal sphincter; *ε*, Hencky strain.

### 2.4 Swallowing Assessments

Each participant underwent a videofluoroscopic swallowing (VFS) study using a Siemens Luminos Agile Max Fluoroscopy unit (Siemens Healthineers), a video x‐ray diagnostic procedure for swallowing disorders, at baseline, 3, and 6 months. All VFS studies were recorded at 30 frames/second on a TIMS DICOM system (Version 3.2, TIMS Medical, TM, Chelmsford, MA). Trials of food and liquid were prepared using FDA‐approved standardized barium contrast agents (Varibar) according to the International Dysphagia Diet Standardization Initiative (IDDSI) [24, 25]. Each VFSS included two trials of each of the following bolus types: 5 mL of thin liquid (IDDSI level 0); 10 mL of thin liquid (IDDSI Level 0); cup sips of thin liquid (IDDSI Level 0); 5 mL of pudding (IDDSI Level 4); and bite sized crackers with 3 mL of pudding (IDDSI Level 7) on top. For the purposes of this study, trials of thin liquid sips via cup (IDDSI Level 0) and 5 mL of pudding (IDDSI Level 4) were evaluated.

Frame‐by‐frame analysis of recordings was completed using Image J software [26] for the following metrics: DIGEST [27], swallowing temporal measures [28], and %(C2–C4)^2^ pharyngeal residue [29]. DIGEST is a validated framework that grades dysphagia severity based on swallowing safety (airway protection) and efficiency (pharyngeal clearance), with an overall composite grade [27]. Temporal swallowing events were analyzed to characterize timing measures, and pharyngeal residue was quantified as pixel‐based %(C2–C4)^2^—representing the percentage of residue with respect to the individual's cervical spine [29]. Two trained raters achieved ≥ 80% inter‐ and intra‐rater reliability prior to analyzing VFS studies. All VFS study recordings were scored independently by these two raters; discrepancies were resolved by consensus. Patient‐reported swallowing burden was measured using the Eating Assessment Tool (EAT‐10 [30]) across all timepoints.

### 2.5 Salivary Assessments

Stimulated saliva was collected using a metronome‐guided chewing protocol (70 beats per minute) over 5 minutes. Participants simulated chewing to the metronome beat and expectorated into a tube when significant amounts of saliva had pooled in their mouths. Saliva was kept on ice following collection and, within 4 h of collection, saliva was analyzed for flow rate (mL/min using weight on an OHAUS Pioneer PX163/E scale), pH (Orion Star A211 pH meter, Thermo Fisher Scientific), and extensional viscosity (Capillary Breakup Extensional Rheometer (CaBER), Thermo Fisher Scientific) [31, 32, 33, 34]. Flow rate (mL/min) quantified the amount of salivary output to assess for hyposalivation. Extensional viscosity—a measure of a fluid's resistance to an extensional flow, relevant for surface and bolus cohesion and lubrication—was measured. Three boluses were loaded into the CaBER, each run four times; data from the first viable run were included in analyses. Salivary extensional viscosity was derived at Hencky strain rates from 9.0 to 10.0, where the data were most stable and complete across participants. Salivary breakup time, representing the time required for a stretched salivary filament to thin and break under force, was also recorded.

### 2.6 Statistical Analysis

DIGEST grades, temporal, and pharyngeal residue measures were compared to normative values using one‐sample Wilcoxon signed‐rank tests. Forest plots were used to visualize median, 2.5, and 97.5 percentiles of pharyngeal residue and temporal measures compared to normative values [35]. Comparisons with normative values were performed using two‐sample Mann–Whitney *U* tests for EAT‐10 scores and one‐sample *t*‐tests for stimulated salivary flow rates.

For within‐subjects analyses, longitudinal changes in all outcomes were analyzed using generalized estimating equation (GEE) models with exchangeable correlation structures. Spearman's rho was used to examine associations between changes in swallowing and salivary measures and changes in EAT‐10 scores from baseline to 3 months. Univariable proportional odds models assessed temporal measures as predictors of DIGEST safety and efficiency grades at 3 months. All analyses were conducted in SAS 9.4.1 (Cary, NC).

## 3 Results

Sixteen participants were enrolled in the original study [23]; of these, 12 were eligible for CLR 131 treatment. Those same 12 participants were eligible for this secondary analysis. Ten of the 12 participants had prior chemotherapy and/or RT at least 1 year before study enrollment. On average, baseline data was gathered 2.2 months (range: 1.60–5.37 months) following recurrence diagnosis. The mean time from completion of initial treatment to recurrence diagnosis was 3.45 years (range: 0.44–11.11 years). Participant demographics are presented in Table 2.

**TABLE 2:** Participant demographics at baseline.

Characteristic	Total patients (*N* = 12)
*Sex*	
Female^a^	3 (25%)
Male	9 (75%)
*Age*	
Median age at baseline (range)	65.5 (47–84)
*Ethnicity*	
White	12
Other	0
Hispanic	0
Non‐Hispanic	12
*ECOG performance status*	
ECOG 0	9 (75%)
ECOG 1	3 (25%)
*Primary tumor site*	
Oropharynx	5 (42%)
p16 positive	4 (80%)
p16 negative	1 (20%)
Nasopharynx	1 (8%)
Larynx	1 (8%)
Oral cavity	4 (33%)
Salivary gland	1 (8%)
*T stage (AJCC 8th ed.)*	
T1–T2	6 (50%)
T3–T4	6 (50%)
*N stage (AJCC 8th ed.)*	
N0–1	6 (50%)
N2–3	6 (50%)
*AJCC stage (8th ed.)*	
I	2 (17%)
II	3 (25%)
III	3 (25%)
IVA	2 (17%)
IVB	2 (17%)
*Initial treatment at diagnosis*	
Surgical resection	7 (58%)
No adjuvant treatment	1 (8%)
Adjuvant radiation	4 (33%)
Adjuvant chemoradiation	2 (17%)
Definitive chemoradiation	5 (42%)
*Recurrence status at study entry*	
First recurrence^b^	6 (50%)
Multiply recurrent	6 (50%)
Metastatic disease^b^	1 (8%)

Abbreviations: AJCC, American Joint Committee on Cancer; ECOG, Eastern Cooperative Oncology Group.

^a^ Participants were asked to report their biological sex (male/female) through a self‐report questionnaire.

^b^ Participant developed metastatic disease at the first recurrence.

### 3.1 Baseline Swallowing and Salivary Function Compared to Normative Data

At baseline, participants demonstrated significant impairment in swallowing and salivary function relative to normative values (Table 3). DIGEST grades (11 of 12 participants) were significantly worse with reduced safety, efficiency, and overall grades (*p* < 0.0001). Vallecular and pyriform sinus residue (10 of 12 participants) was significantly greater than in healthy individuals (*p* < 0.05). All swallowing temporal measures (10 of 12 participants) were significantly prolonged for both boluses of cup sip of thin liquid and 5 mL of pudding (*p* < 0.001), with the exception of hyoid burst to UES opening with cup sip of thin liquid. Participants also reported greater perceived swallowing difficulty, with mean EAT‐10 scores significantly higher compared to age‐matched healthy individuals [36] (*p* < 0.0001); 72.4% of participants scored ≥ 3 (abnormal results, warranting additional swallowing assessment), compared to 10.5% of healthy adults [36]. Stimulated salivary flow rate (11 of 12 participants) was significantly lower than normative values [37] (*p* < 0.0001). Forest plots of pharyngeal residue and temporal measures are shown in Figure 1. Salivary extensional viscosity data could not be compared to normative data due to a lower capture rate than in published healthy studies.

**TABLE 3:** Swallowing and salivary measures compared to normative data.

Swallowing measures					
		Prior RT		Normative	*p*
		*N*_T_	Median (IQR)	Median	
Cup sip of thin	Swallow reaction time	30	283 (50, 533)	133	< 0.001^a^
	Hyoid burst to UES opening	23	133 (67, 150)	100	0.791^a^
	UES opening duration	23	567 (500, 650)	467	< 0.001^a^
	Time to LVC	23	317 (233, 367)	133	< 0.001^a^
	LVC duration	23	717 (517, 1517)	467	< 0.001^a^
5 mL of pudding	Swallow reaction time	22	900 (117, 2167)	367	< 0.001^a^
	Hyoid burst to UES opening	22	233 (200, 267)	167	< 0.001^a^
	UES opening duration	22	500 (417, 583)	400	< 0.001^a^
	Time to LVC	22	283 (233, 317)	133	< 0.001^a^
	LVC duration	22	508 (433, 550)	434	< 0.001^a^

		*N*_T_	Median (IQR)	Normative value	*p*
	DIGEST safety	24	1 (1, 2)	0	< 0.0001^a^
	DIGEST efficiency	24	3 (0, 3)	0	< 0.0001^a^
	DIGEST overall	24	2 (1, 3)	0	< 0.0001^a^

		*N*_T_ (%)		*N* (%)	p
	EAT‐10 Score < 3	8 (27.6)		34 (89.5)	*p* < 0.0001^b^
	EAT‐10 Score ≥ 3	21 (72.4)		4 (10.5)	*p* < 0.0001^b^

Salivary measures					
		*N*_T_		*N*	*p*
	Stimulated salivary flow rate	26		50	*p* < 0.0001^c^

Abbreviation: *N*_T_, number of trials.

^a^ Measures compared using one‐sample signed rank test.

^b^ Measures compared using two‐sample Mann–Whitney *U* test.

^c^ Measures compared using one‐sample *t*‐test.

**FIGURE 1:**
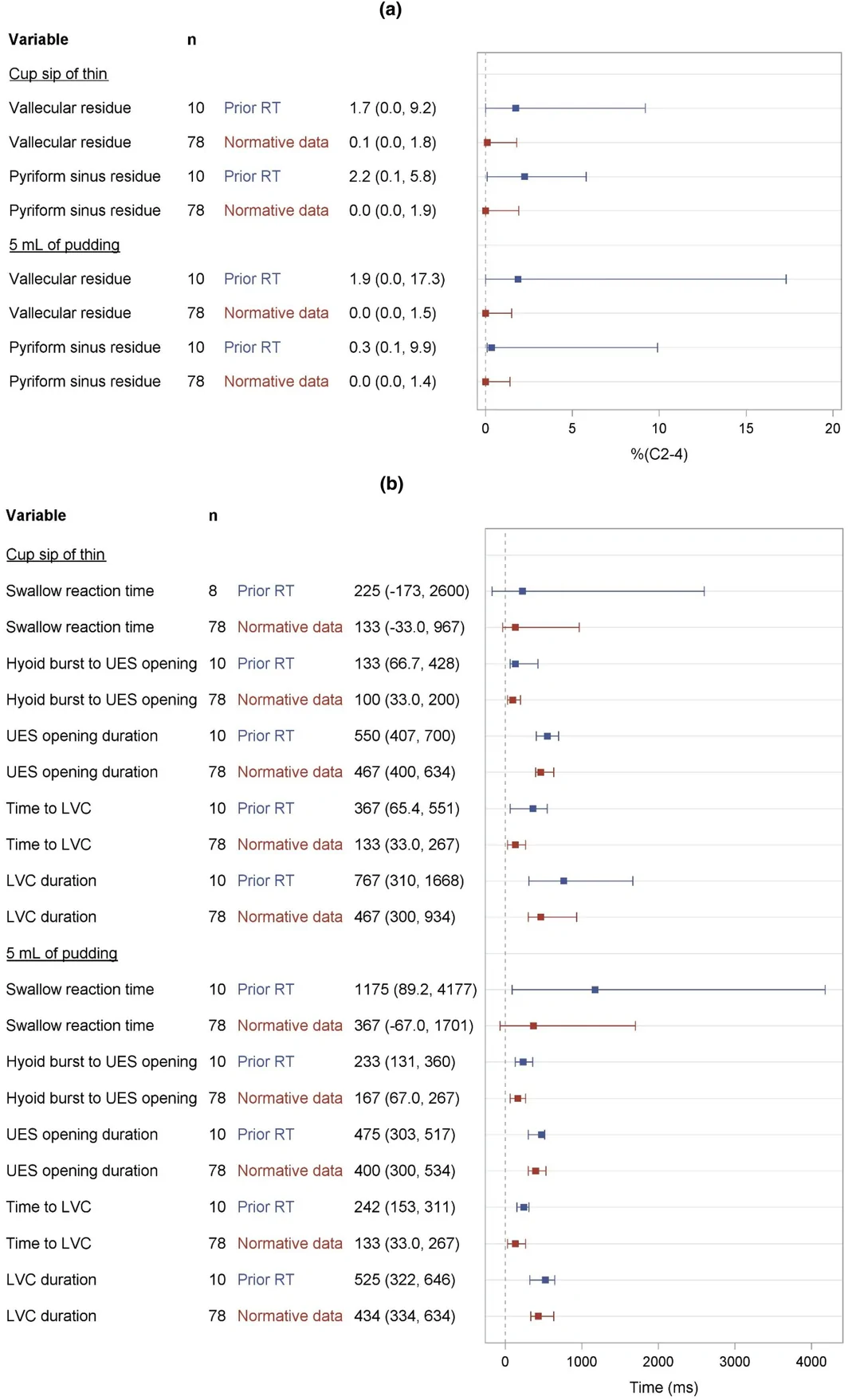
Forest plots of pharyngeal residue and temporal measures of swallowing compared to normative data. (A) Baseline pharyngeal residue: Median (2.5%ile, 97.5%ile). (B) Baseline swallowing temporal measures: Median (2.5%ile, 97.5%ile). [Color figure can be viewed at wileyonlinelibrary.com]

### 3.2 Longitudinal Changes of Swallowing and Salivary Function After CLR 131 and EBRT

From the existing data present, most swallowing measures remained stable over time. It should be noted that while 12 participants were assessed at baseline, follow‐up data were available for 10 participants at 3 months post‐treatment, 6 participants at 6 months, and 2 participants at 12 months. The only significant swallowing change was longer time to LVC (the time it takes from the start of the swallow to complete laryngeal vestibule closure) with 5 mL of pudding at 3 months (*p* < 0.02). However, salivary profiles showed more dynamic changes. Stimulated salivary flow rate declined significantly at 12 months (*p* < 0.0001); salivary pH decreased significantly at 3 months compared to baseline (*p* < 0.05). Salivary extensional viscosity increased at 6 months at 9.0–9.5 Hencky strain (*p* < 0.0001) and at both 3 and 6 months at 9.5–10.0 Hencky strain (*p* < 0.002; *p* < 0.0001), but declined at 12 months at 9.0–9.5 Hencky strain (*p* < 0.0001). EAT‐10 scores increased significantly at 6 months (*p* < 0.02). These changes are depicted in Figure 2a–f.

**FIGURE 2:**
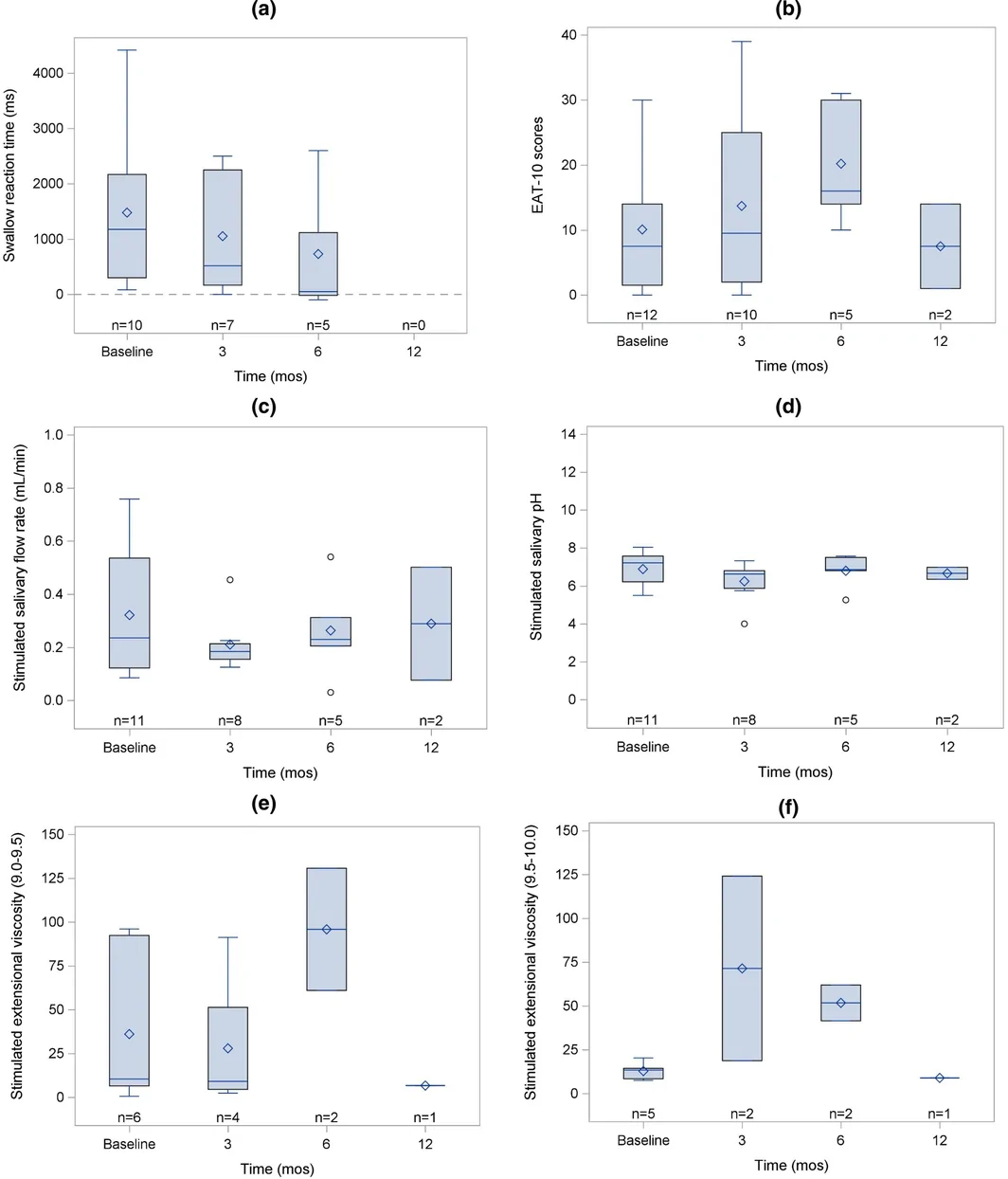
Swallowing and salivary measures at each assessment point. (a) Time to LVC with 5 mL of pudding at each assessment point. (b) EAT‐10 scores at each assessment point. (c) Stimulated salivary flow rate at each assessment point. (d) Stimulated salivary pH at each assessment point. (e) Stimulated extensional viscosity (9.0–9.5) at each assessment point. (f) Stimulated extensional viscosity (9.5–10.0) at each assessment point. [Color figure can be viewed at wileyonlinelibrary.com]

### 3.3 Associations Among Swallowing and Salivary Outcomes and Perceived Dysphagia Burden

From baseline to 3 months, several moderate‐to‐strong correlations developed between swallowing and salivary characteristics and EAT‐10 scores. Time to LVC with 5 mL of pudding had a moderate negative correlation with EAT‐10 scores (*ρ* = −0.60), such that decreased time to LVC tended to associate with increased EAT‐10 scores and vice versa. Salivary pH showed a strong positive correlation with EAT‐10 scores (*ρ* = 0.84), while extensional viscosity showed a strong negative correlation at 9.0–9.5 and 9.0–10.0 Hencky strains (*ρ* = −0.99). Salivary breakup time demonstrated a moderate positive correlation with EAT‐10 scores (*ρ* = 0.57). These are shown in Figure 3a–c.

**FIGURE 3:**
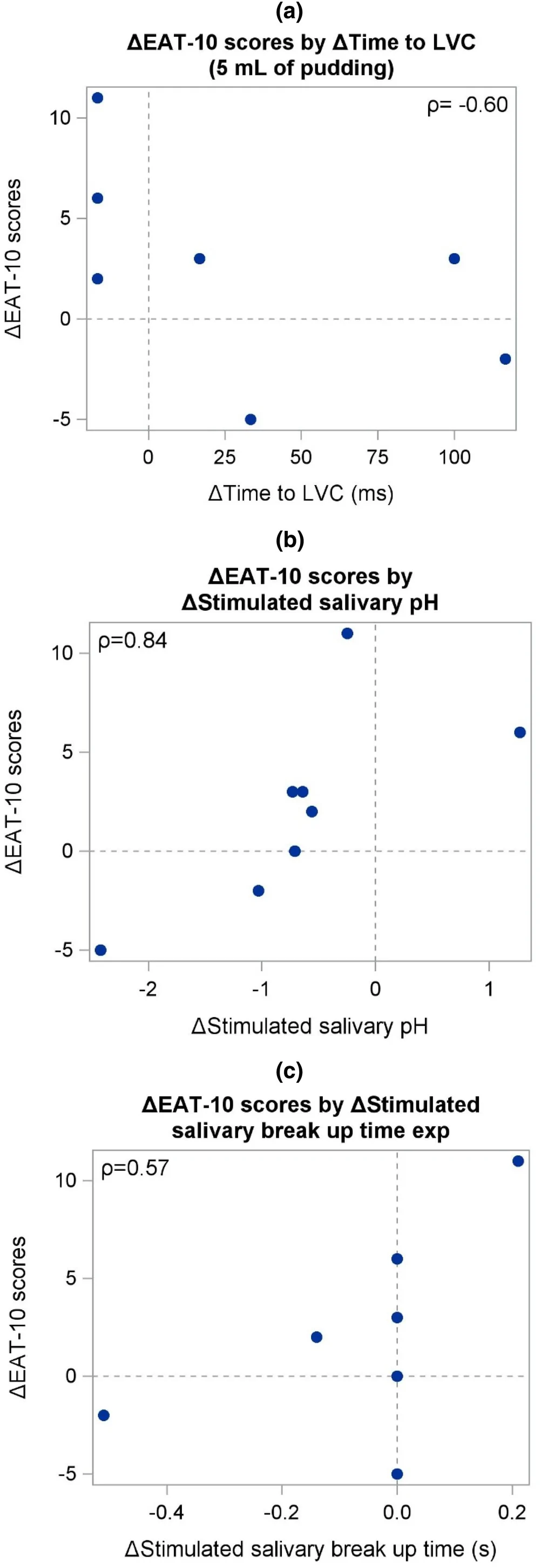
(a) ΔEAT‐10 scores by ΔTime to LVC from baseline to 3 months. (b) ΔEAT‐10 scores by ΔStimulated salivary pH from baseline to 3 months. (c) ΔEAT‐10 scores by ΔStimulated salivary break up time from baseline to 3 months. [Color figure can be viewed at wileyonlinelibrary.com]

### 3.4 Predictors of DIGEST Safety and Efficiency Grades

No swallowing temporal measures significantly predicted DIGEST safety or efficiency grades at 3 months post‐treatment.

## 4 Discussion

As novel radiotherapeutics such as CLR 131 are increasingly integrated into treatment protocols for recurrent HNC, it is important to understand their functional consequences beyond tumor control. Dysphagia and xerostomia are well‐recognized long‐term toxicities affecting survivorship in this population. This study provides a preliminary characterization of swallowing physiology and salivary changes following CLR 131 combined with EBRT. These findings contribute to the emerging toxicity profile of this treatment approach and may inform multidisciplinary survivorship care, including salivary assessments and targeted rehabilitation exercises; these data provide an important foundation for future, larger studies evaluating functional outcomes associated with this novel treatment.

This study provides one of the first longitudinal evaluations of swallowing and salivary outcomes in patients with recurrent HNC treated with CLR 131 in combination with EBRT. At baseline, patients demonstrated significant dysphagia and salivary hypofunction, consistent with prior reports of late radiation‐associated dysphagia (late‐RAD) and xerostomia following primary chemoradiation or surgery [38]. These findings highlight the persistent and cumulative nature of treatment‐related toxicity in this population and the clinical challenge of optimizing tumor control while preserving function.

Despite the severe baseline impairments, most instrumental swallowing measures remained stable following CLR 131 and EBRT, suggesting that this treatment combination may not further compromise swallowing physiology in the early post‐treatment period. This stability contrasts with the progressive decline often observed after re‐RT alone and supports the potential tissue‐sparing benefit of CLR 131 as an adjunctive radiotherapeutic agent. However, patient‐reported swallowing burden increased at 6 months, indicating that subjective functional decline may develop earlier than measurable physiologic change. This difference highlights the importance of pairing instrumental assessment with patient‐reported outcomes to comprehensively capture post‐treatment dysphagia.

Salivary measures demonstrated dynamic changes over time, including reduced flow rate and pH and variable extensional viscosity. Interestingly, when examining the relationships among salivary characteristics and perceived swallowing difficulty, higher extensional viscosity in this cohort was associated with lower EAT‐10 scores; this relationship between viscosity and patient‐reported swallowing burden warrants further study, including compositional analyses of salivary components, including mucins that influence salivary rheology. These findings highlight that both the quantity and quality of saliva contribute to perceived dysphagia burden. Swallowing and salivary production are interrelated, as saliva provides essential lubrication to maintain bolus cohesion and facilitate oral and pharyngeal transport. Additionally, salivary composition may impact sensory input and impact swallowing performance [39]. Characterizing the impact of salivary composition on swallowing may inform the development of novel therapeutic approaches for dysphagia. Given this, clinical management of patients with or at risk for dysphagia should include assessment of salivary function beyond quantifying saliva volume alone.

Temporal swallowing measures were prolonged relative to normative data but did not predict DIGEST safety or efficiency grades. These results suggest that timing abnormalities alone may not drive swallowing impairment; other changes in structural displacement, including reduced hyolaryngeal excursion, incomplete epiglottic inversion, or impaired pharyngeal constriction, may also contribute to these functional outcomes. Comprehensive physiological analyses incorporating additional measures are needed to identify the mechanisms most strongly linked to functional outcomes following re‐RT.

Together, these findings suggest that while recurrent HNC patients begin with substantial swallowing and salivary compromise, the addition of CLR 131 and EBRT may not cause significant further deterioration in swallowing and salivary function. However, patient perception of swallowing difficulty remains sensitive to salivary alterations, emphasizing the need for management strategies addressing both salivary and swallowing issues following radiation.

## 5 Limitations and Future Directions

Interpretation of these findings is limited by the small sample size, attrition due to disease progression, inability to identify participants undergoing swallowing therapy, and variability in baseline timepoints relative to initial diagnosis and recurrence. Additional limitations include use of comparison to published literature from healthy cohorts rather than having our own well‐matched comparison group of patients with recurrent HNC, a low capture rate for salivary viscosity data relative to published normative data, and the analysis of only two bolus consistencies administered during the VFS studies. A focus on DIGEST functional outcomes may have limited sensitivity to detect subtle physiologic changes. Future studies should employ larger cohorts, well‐matched comparison groups, longer follow‐up, and comprehensive physiologic metrics to clarify trajectories of improvement or decline. Incorporating salivary composition analyses—including quantification of mucins and other analytes known to influence salivary rheology—will be important for understanding how changes in saliva contribute to swallowing function, patient‐reported outcomes, and quality of life.

## 6 Conclusions

In recurrent HNC, late treatment effects remain a primary contributor to swallowing and salivary impairment. While CLR 131 and EBRT did not exacerbate most swallowing outcomes, patient‐reported burden increased and correlated with salivary changes. These results highlight the interplay between salivary quality and perceived swallowing function and support ongoing investigation of CLR 131 as a potentially less toxic therapeutic adjunct in re‐RT protocols.

## Funding

This work was supported by Specialized Program of Research Excellence, National Cancer Institute, P50CA278595 and National Institute on Deafness and Other Communication Disorders, National Institutes of Health, T32DC009401.

## Data Availability

The data that support the findings of this study are available on request from the corresponding author. The data are not publicly available due to privacy or ethical restrictions.
